# Performance of Cardiotropic rAAV Vectors Is Dependent on Production Method

**DOI:** 10.3390/v14081623

**Published:** 2022-07-26

**Authors:** Renuka Rao, Melad Farraha, Grant J. Logan, Sindhu Igoor, Cindy Y. Kok, James J. H. Chong, Ian E. Alexander, Eddy Kizana

**Affiliations:** 1 Centre for Heart Research, The Westmead Institute for Medical Research, Westmead 2145, Australia; renuka.rao@sydney.edu.au (R.R.); mfar4366@uni.sydney.edu.au (M.F.); sindhu.igoor@sydney.edu.au (S.I.); cindy.kok@sydney.edu.au (C.Y.K.); james.chong@sydney.edu.au (J.J.H.C.); 2 Sydney Medical School, Faculty of Medicine and Health, The University of Sydney, Sydney 2006, Australia; glogan@cmri.org.au; 3 Gene Therapy Research Unit, Children′s Medical Research Institute and Sydney Children’s Hospitals Network, Faculty of Medicine and Health, The University of Sydney, Westmead 2145, Australia; ian.alexander@health.nsw.gov.au; 4 Department of Cardiology, Westmead Hospital, Westmead 2145, Australia; 5 Discipline of Child and Adolescent Health, Faculty of Medicine and Health, The University of Sydney, Sydney 2006, Australia

**Keywords:** baculovirus, gene therapy, rAAV vectors, vector production, viral vectors

## Abstract

Gene therapy is making significant impact on a modest, yet growing, number of human diseases. Justifiably, the preferred viral vector for clinical use is that based on recombinant adeno-associated virus (rAAV). There is a need to scale up rAAV vector production with the transition from pre-clinical models to human application. Standard production methods based on the adherent cell type (HEK293) are limited in scalability and other methods, such as those based on the baculovirus and non-adherent insect cell (Sf9) system, have been pursued as an alternative to increase rAAV production. In this study, we compare these two production methods for cardiotropic rAAVs. Transduction efficiency for both production methods was assessed in primary cardiomyocytes, human induced pluripotent stem cell-derived cardiomyocytes (hiPSC-CMs), and in mice following systemic delivery. We found that the rAAV produced by the traditional HEK293 method out-performed vector produced using the baculovirus/Sf9 system in vitro and in vivo. This finding provides a potential caveat for vector function when using the baculovirus/Sf9 production system and underscores the need for thorough assessment of vector performance when using diverse rAAV production methods.

## 1. Introduction

Recombinant adeno-associated viral (rAAV) vectors are currently being employed for clinical trials as the preferred gene therapy vehicle. Depending on the serotype, they exhibit tropism for specific tissues making them suitable for systemic delivery [1,2]. The classical method of production for small scale applications is the triple plasmid transfection of human epithelial kidney (HEK) 293 cells. One of the major obstacles for clinical application of this vector system is large scale production. Different systems have been trialed for scaling up production including transfection of HEK293 cells grown in suspension [3], production through stable cell lines [4], or use of the herpes simplex virus as a vector for rAAV production by mammalian cells [5], all with limited success. Baculovirus-based production of rAAV using three Bacmids (BAC) developed by the Kotin group [6] offered an attractive alternative. However, the success of this system was limited mostly to rAAV2 based vectors. The system went through additional improvements including reduction in the number of coinfecting BACS by combining the rep and cap into one BAC resulting in the development of the two BAC system [7]. Further developments consisted of using stable insect Sf9 cell lines harbouring silent copies of rAAV rep and cap genes for production of the different serotypes (rAAV1–12), known as the one BAC system [8]. Additional advances included increasing relative viral capsid proteins (VP) 1 and 2 content, based on a complex modification of the Kozak sequence preceding the VP1 start codon [9]. This approach required fine adjustment of the VP1 Kozak sequence in the narrow window of the translation initiation site (TIS) variable effect and dependency on rAAV serotype. Thus, the optimal TIS needs to be empirically identified for each serotype.

For cardiac gene therapy, rAAV serotypes 1, 6, and 9 have emerged as the most tropic vectors [10]. Recombinant AAV9 has proven to be the most efficient at transducing mouse and rat cardiomyocytes, when delivered systemically [10]. Knowledge regarding cardiac tropism of rAAVs in large animals, however, is limited with some indication that serotypes 6 and 9 are capable of transducing cardiomyocytes more efficiently than other serotypes [11]. Several laboratories have had success with cardiac gene therapy using rAAV9 for rodent models of cardiac disease [12]. As a pre-requisite for human studies, most regulatory authorities require demonstration of safety and efficacy in large animal models. The quantity of rAAV vector necessary for this work, however, requires significant scale up in rAAV vector production. Hence, this study was undertaken to determine the feasibility of vector production for the cardiotropic rAAV serotypes 2/6, 2/8, and 2/9 using the two BAC insect cell baculoviral derived system [7] and performance compared to the standard HEK 293 production methods for cardiac transduction in vitro and in vivo.

## 2. Materials and Methods

### 2.1. rAAV Vector Production in HEK293 Cells

Vectors were prepared with either the two or three plasmid systems based on the capsid serotype, as previously described with modifications [13,14,15]. The gene of interest vector encoded enhanced green fluorescent protein (GFP) with the wild-type woodchuck post transcription regulatory element (WPRE) under the control of the chicken beta actin (CBA) promoter–referred to as the AAV-CBA-GFP-WPRE plasmid. rAAV was produced as described by Farraha et al. [15]. See Supplementary Materials for details.

### 2.2. rAAV Vector Production in Sf9 Cells

#### 2.2.1. Plasmid Constructs

Plasmid with gene of interest: The plasmid pFBGR containing GFP gene under the dual control of the cytomegalovirus IE promoter (CMV) and baculovirus p10 promoter (p10), flanked by AAV2 inverted terminal repeats (ITR), was obtained from Dr. Rob Kotin’s laboratory (NIH, Bethesda, MD, USA). The mammalian GFP expression cassette in pFBGR was replaced with the expression cassette from plasmid AAV-CBA-GFP -WPRE by sub-cloning into the PstI restriction site.

Rep-cap plasmids: The plasmid pSR660 containing AAV2 rep gene in combination with an ACG initiated AAV8 cap expression cassette was obtained from Dr Rob Kotin’s laboratory. The plasmid pSR646 containing AAV2 rep gene in combination with a non-canonical start TTG codon containing AAV6 cap was purchased from Addgene. The codon-optimized plasmid pSR660 (AAV9 serotype) was generated by replacing the AAV-Cap8 cassette with a PCR amplified 2.2 Kb AAV-Cap9 into the Nhe1/Xma1 site of pSR660 (AAV8 serotype).

#### 2.2.2. Generation and Production of Recombinant Baculoviral Stocks

Plasmids described above were transformed into DH10Bac-competent cells to generate recombinant bacmids in accordance with the manufacturer’s protocol (Invitrogen, Carlsband, CA, USA). The pBAC genomes generated were transfected with cellfectin transfection reagent (Thermo Fisher Scientific, Waltham, MA, USA) into the Sf9 insect cell line to produce rBAC-Rep2/Cap6, rBAC-Rep2/Cap8, rBAC-Rep2/Cap9, and rBAC-GFP recombinant baculoviral vectors.

Sf9 cells were grown in suspension cultures in SF-900 II SFM media (Life Technologies, Carlsbad, CA, USA) without any serum supplementation. All four rBAC clones were amplified through three consecutive infectious passages in Sf9 cells. Recombinant baculoviral vectors were stored as P3 stocks at 4 °C in the dark supplemented with 1% FBS. Baculoviral stocks were titrated by flow cytometry, measuring the baculoviral envelope (gp64) protein. A titre of around 10^8^ vg/mL was obtained routinely for the P3 stocks. Aliquots of P3 stocks for all the above bacmids were stored at−80 °C.

Production of rAAV in sf9 cells was performed with some modifications [16,17,18] as described in the Supplementary Materials.

### 2.3. Cell Culture

The highly AAV-permissive 2V6.11 cells (CRL-2784, ATCC, Manassas, VA, USA) [19] were plated at a density of 2 × 10^5^ cells/well in the presence of ponesterone at 1 µg/mL in a 24 well plate. The next day, cells were counted and based on the cell density; rAAV vector was added at a MOI of 15,000 per well. Twenty-four hours later, the media was replaced with DMEM containing 2% FBS. Forty-eight hours post transduction, the cells were imaged using a Zeiss Axiovert 200 M live-cell imaging microscope (Zeiss, Oberkochen, Germany), and analysed for GFP expression, using flow cytometry on a BD FACS Canto II (BD Biosciences, Franklin Lakes, NJ, USA). To determine the dose response to rAAV, cells were transduced with both HEK293 and baculovirus expression vector (BEV) system produced rAAV at MOIs of 2500, 5000, and 10,000.

### 2.4. Neonatal Rat Ventricular Myocytes (NRVMs)

NRVMs were isolated from day 2–3 neonatal rat hearts as previously described [20]. See Supplementary Materials for details.

For comparative transduction efficiency, BEV and HEK293-derived rAAV6-GFP was added to the cells at an MOI of 30,000. Six days post transduction, the cells were imaged using a Zeiss Axiovert 200 M live-cell imaging microscope (Zeiss, Oberkochen, Germany), and analysed for GFP expression, using flow cytometry on a BD FACS Canto II (BD Biosciences, Franklin Lakes, NJ, USA).

### 2.5. Human Induced-Pluripotent Stem Cell-Derived Cardiomyocytes (hiPSC-CMs)

Transduction efficiency was also determined for HEK 293 and BEV derived rAAV6-GFP using hiPSC-CMs. Maintenance of undifferentiated cells and differentiation was undertaken as previously described [21,22]. See Supplementary Materials for details. hiPSC-CMs were plated at 2 × 10^5^ cells/well and transduced with rAAV6-GFP at an MOI of 30,000. Six days post transduction, the cells were imaged using a Zeiss Axiovert 200 M live-cell imaging microscope (Zeiss, Oberkochen, Germany), and analysed for GFP expression, using flow cytometry on a BD FACS Canto II (BD Biosciences, Franklin Lakes, NJ, USA).

### 2.6. Flow Cytometry

Cells were washed once with 500 µL of PBS. Cells were harvested using 250 µL of tryple express (Thermo Fisher Scientific, Waltham, MA, USA) per well and incubated at 37 °C for 10 min. Cells were then dissociated and collected in eppendorf tubes. Wells were washed with cold PBS supplemented with 2% FBS and pooled with previous collections in eppendorf tubes. Cells were centrifuged at 3500 rpm for 5 min and washed once with 500 µL of PBS with 2% FBS. Cells were then resuspended in 400 µL of DAPI at 100 ng/mL in cold PBS and analysed using the BD FACS Canto II (BD Biosciences, Franklin Lakes, NJ, USA). The gating strategy is shown in Figure S2.

### 2.7. Western Blot Analysis for VP Proteins

VP protein composition for the various rAAV serotypes produced by HEK293 and BEV systems was determined by resolving 1 × 10^11^ vector genomes on 4–12% bis-tris polyacrylamide gels (Novex, Invitrogen, Carlsband, CA, USA). Following gel electrophoresis, proteins were transferred onto polyvinylidene difluoride (PVDF) membranes and incubated with a monoclonal anti VP1 + VP2 + VP3 antibody (10R-A114A, Fitzgerald, Crossville, TN, USA) at a dilution of 1:1000 at 4 °C overnight. The blots were then incubated with a secondary anti-mouse IgG (whole molecule) antibody labelled with peroxidase at a dilution of 1:10,000 (Sigma–Aldrich, St. Louis, MO, USA) for 1 h at room temperature. Membranes were washed with TBS-T (10 mM Tris-HCl [pH 7.6], 0.15 M NaCl, 0.05% Tween 20) thrice for 5 min each after primary and secondary antibody incubations. Detection was carried out using the ECL Western Detection Kit (Biorad, Hercules, CA, USA).

To check for the presence of cathepsin degradation products, Western blots were imaged using the odyssey system. In brief, PVDF membranes were incubated with anti VP1 + VP2 + VP3 rabbit polyclonal antibody (03-61084, American Research Products Inc. ^TM^, Waltham, MA, USA) at 1:500 dilution at 4 °C overnight. Blots were then incubated with secondary goat anti-Rabbit antibody (LICOR, Lincoln, NE, USA) on a shaker for 1 h at room temperature in the dark. Membranes were washed with PBS-T (PBS, 0.1% Tween 20) four times for 5 min each. Blots were then rinsed with PBS and imaged on the odyssey system using software image studio version 5.2 (LICOR, Lincoln, NE, USA).

### 2.8. AAV Vector Performance in Mice

**Figure 1:**
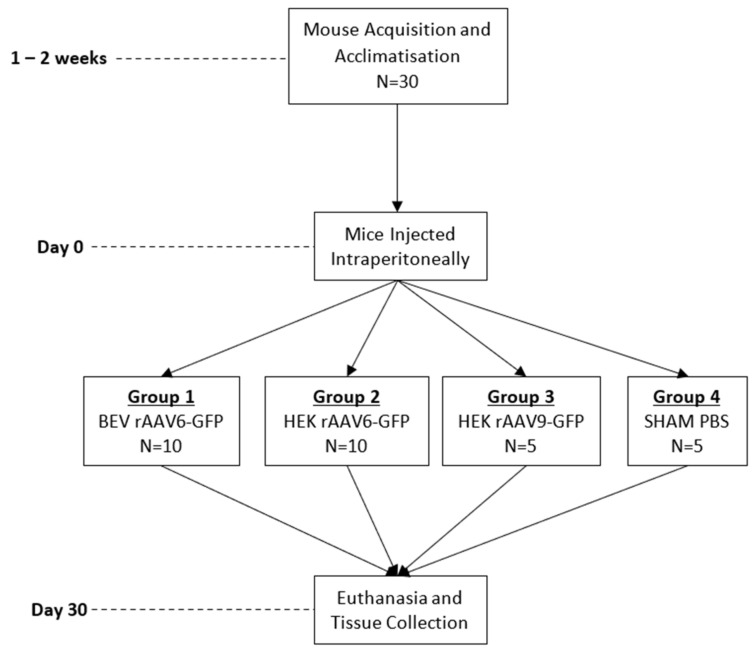
Summary of in vivo experiment timeline and animal group allocation.

C57BL/6 mice (male, 6 weeks old) were injected via an intraperitoneal route with a dose of 1 × 10^11^ vg/mouse. In total, four groups of mice were injected as follows: HEK293 rAAV6-GFP, BEV rAAV6-GFP, HEK293 rAAV9-GFP (positive control), and a negative control group was injected with PBS (Figure 1). Groups 1 and 2 had ten mice each while groups 3 and 4 had five mice each. Four weeks post injection, mice were euthanised and heart, liver, lung, diaphragm, and muscle tissues were harvested for genomic DNA and RNA extractions.

### 2.9. Genomic DNA Extraction and Vector Genome Copy Number Analysis

Total genomic DNA was extracted from frozen tissue using the DNeasy blood and tissue kit (Cat. no. 69504, Qiagen, Hilden, Germany). An amount of 200 ng triplicates of each sample were subjected to qPCR using a primer probe specific for GFP present in the rAAV-GFP vector genome construct (Table S1) with the Sensifast Probe, No ROX Kit (Cat no BIO-86020, Bioline, TN, USA. The number of vector DNA copies in each sample was calculated using a standard curve generated from serial dilutions of a plasmid containing the qPCR target sequence. qPCR was performed in a 384 well plate in a Biorad CFX 384 PCR thermocycler (Biorad, Hercules, CA, USA). Statistical analysis was performed using prism software v8 (Graphpad, San Diego, CA, USA).

### 2.10. RNA Extraction and GFP Expression Via qPCR

RNA was extracted from liver tissue using the isolate II RNA mini kit (Cat. no. BIO-52073, Bioline, TN, USA) and from cardiac tissue using the RNeasy micro-RNA extraction kit (Cat. no. 74004, Qiagen, Hilden, Germany). An amount of 1µg and 500 ng of RNA from liver and heart tissue, respectively, was subjected to cDNA synthesis. Transgene expression was quantified via RT-qPCR using mouse GFP and ribosomal protein large P0 primers (Rp1p0) (Table S2) with the SensiFast SyBr No ROX kit (Cat. no. BIO-98050, Bioline, TN, USA). Statistical analysis was performed using prism software v8 (Graphpad, San Diego, CA, USA).

## 3. Results and Discussion

### 3.1. Comparison of rAAV Produced by HEK293 and BEV Systems

**Figure 2:**
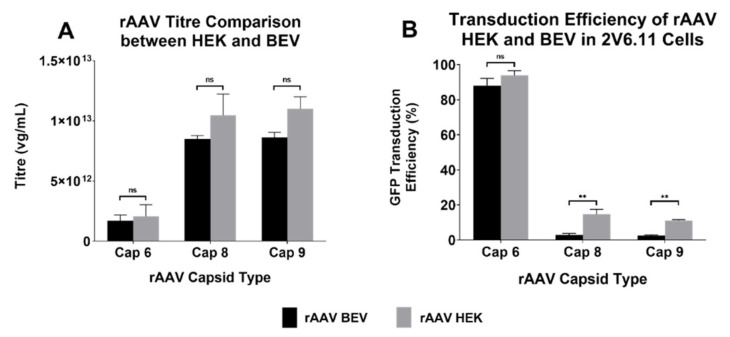
Yields and transduction efficiencies of different serotypes of rAAV produced by HEK293 and BEV systems. (**A**) The titres were quantified via qPCR on the concentrated rAAV samples using WPRE primer probes. rAAV titres of three experiments are displayed as mean ± standard error of mean (SEM). Please note logarithmic scale of the *y*-axis. (**B**) Transduction efficiencies of rAAV-GFP vectors of the three serotypes derived from either HEK293 or BEV were compared. The highly AAV-permissive 2V6.11 cells were transduced with rAAV-GFP vectors at an MOI of 15,000 each. At 48 h post infection, cells were harvested, and the percentage of GFP-positive cells were quantified via flow cytometry analysis. Data pooled from three different experiments are displayed as mean ± SEM. ** *p* ≤ 0.01, ns: non-significance.

In this study, we sought to explore the potential of the baculoviral insect system for scale up of rAAV production. Tamas et al. [7] introduced the 2 BAC system wherein a noncanonical VP1 ORF and an attenuated baculovirus driving Rep78 expression led to 1:1:10 ratio of VP1, VP2, and VP3 proteins. Rep-cap plasmids with similar noncanonical start sites for serotypes 6, 8, and 9 were used for virus production in this study. rAAV6, rAAV8, and rAAV9 vectors produced by the HEK293 and BEV systems had similar PCR titres, with titres for serotypes 8 and 9 being in the range of 1–2 × 10^13^ vg/mL and serotype 6 being 10-fold less, at 1–2 × 10^12^ vg/mL. Thus, irrespective of the production system, titres for all serotypes did not significantly differ (Figure 2A).

The functionality of the vector was also examined. 2V6.11 cells were transduced with the three rAAV serotypes produced by both systems and transduction efficiency for GFP expression was determined via flow cytometry. While HEK293-derived rAAV2/8 and rAAV2/9 were capable of transducing 2V6.11 cells better than their BEV counterparts, BEV-derived rAAV2/6 had similar efficiency as the HEK293-derived rAAV2/6 (Figure 2B).

### 3.2. Characterisation of Capsid Proteins for the Various rAAV Serotypes

**Figure 3:**
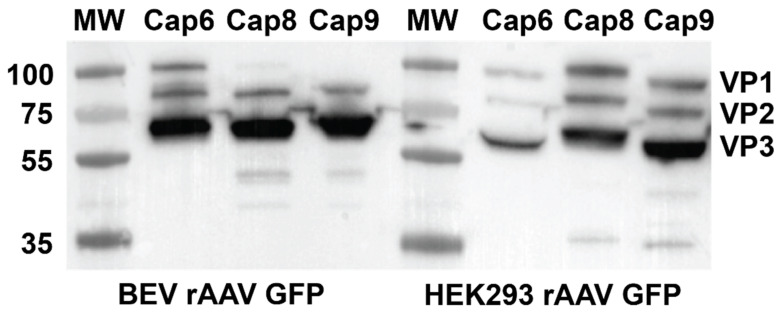
Western blot showing VP1, VP2, and VP3 in rAAV preparations produced in the HEK293 and BEV systems. Viral preps from three independent rAAV preparations for each serotype were analysed by quantitative western blot analysis to compare the ratios of VP1, VP2, and VP3 expressed in HEK 293 or Sf9 cells. VP proteins expressed was analysed by anti VP1 + VP2 + VP3 rabbit polyclonal primary antibody and a secondary anti mouse IgG (whole molecule) antibody labelled with peroxidase and detected using the ECL western detection kit.

To determine the reason for difference in transduction efficiencies with rAAV8 and 9 serotypes, western blot analysis was performed to analyse the composition of VP proteins in the various capsids. While the expected stoichiometry ratio for VP1:VP2: VP3 was maintained in HEK293-derived rAAV8 and 9, VP1 protein was not expressed in the BEV derived rAAV8 and 9 (Figure 3). All preparations of BEV derived rAAV6, however, expressed VP1:VP2:VP3 at a similar stoichiometry ratio as seen with the HEK 293-derived rAAV6. This may partly explain the equivalence of transduction efficiency between HEK293 and BEV rAAV6 in 2V6.11 cells (Figure 3). Thus, as documented in previous studies [23], and as demonstrated by our study, variability in transduction efficiency of BEV derived rAAV is strongly serotype dependent.

### 3.3. Comparison of rAAV6-GFP Transduction Efficiency in Cardiomyocytes

**Figure 4:**
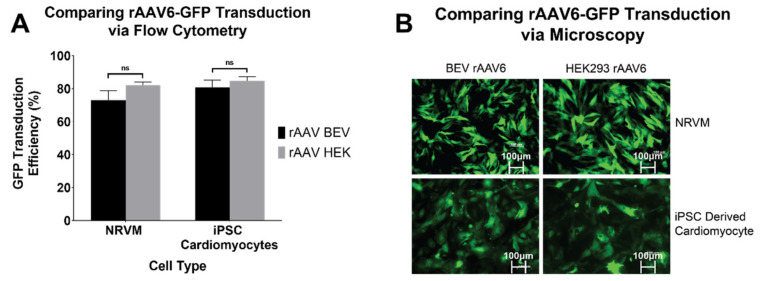
rAAV6-GFP transduction in cardiomyocytes of rodent (NRVM) and human (hiPSC-CM) origin. Transduction efficiencies of rAAV6 serotype were compared in NRVMs and hiPSC-CMs. Cells were transduced at an MOI of 30,000 each with three independent rAAV6 stocks prepared from HEK293 or Sf9 cells. Six days post transduction, cells were harvested and the percentage of GFP positive cells were determined by (**A**) flow cytometry. Results of three independent experiments are displayed as mean ± SEM. and (**B**) fluorescence microscopy. Representative images of GFP expression under a fluorescence microscope, 6 days post transduction. ns: non-significance.

To determine if BEV derived rAAV6 was as efficient as HEK293-derived rAAV6 at transducing relevant cardiac cell types, NRVMs and hiPSC-CMs were assessed. Following transduction, cells were analysed for GFP expression via flow cytometry. No significant difference was observed in GFP expression between BEV and HEK293-derived rAAV6-GFP, respectively, in NRVMs (73 ± 5.7% and 82 ± 1.8%) and hiPSC-CMs (81 ± 4.4% and 85 ± 2.5%) (Figure 4A). Live cell imaging (Figure 4B) verified that BEV and HEK293-derived rAAV6-GFP had the same transduction efficiency. Thus, as previously reported, HEK293 and BEV derived rAAV6 were found to be equally efficient at transducing cardiomyocytes in vitro [9]. This result therefore encouraged experiments to determine the transduction efficiency of BEV derived vectors in vivo.

### 3.4. Comparison of rAAV6-GFP Transduction Efficiency In Vivo

**Figure 5:**
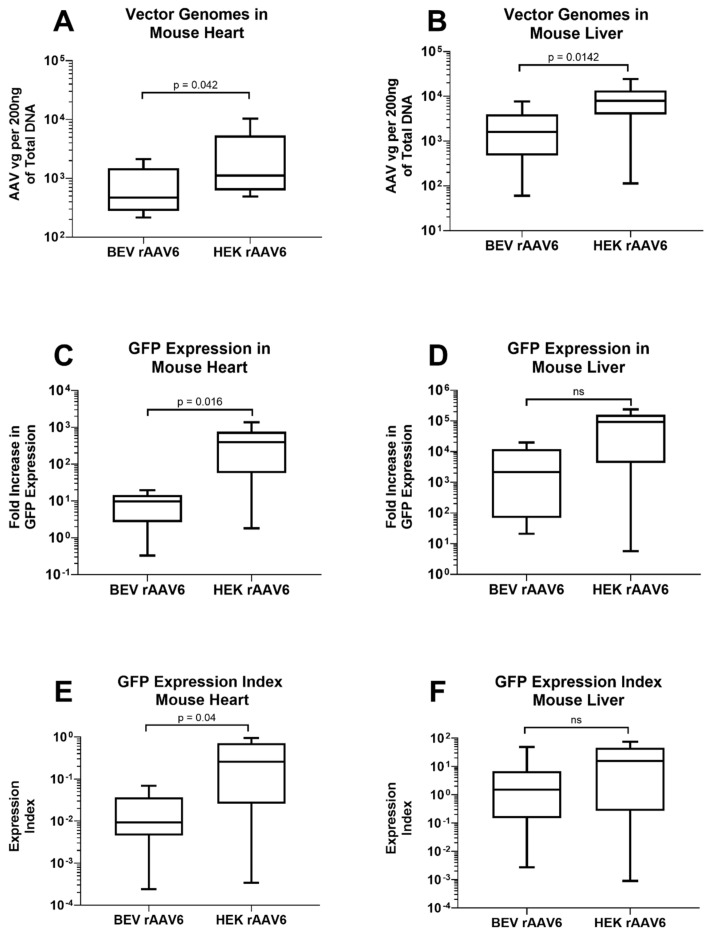
rAAV6-GFP expression in the heart and liver of mice injected with either HEK293 or Sf9 derived rAAV. 200 ng genomic DNA from (**A**) the heart and (**B**) liver were subjected to qPCR for analyses of vector genome copies. RNA from (**C**) the heart and (**D**) liver were converted to cDNA and GFP expression was determined by RT-PCR. Expression index was calculated as ratio of GFP expression normalised to vector genomes for (**E**) heart and (**F**) liver samples (*n* = 9). ns: non-significance.

To compare the transduction potential of BEV and HEK293-derived rAAV6-GFP in vivo, 1 × 10^11^ vg of each vector was injected via an intraperitoneal route into C57BL/6 mice. qPCR for vector genome copies revealed that rAAV vector genomes were consistently higher in the liver, heart, lung, and muscles of mice that received HEK293-derived rAAV6-GFP compared to the mice that received BEV derived rAAV6-GFP. The hearts from mice treated with HEK293-derived vector contained on average 2969 ± 1123 vg compared to 810 ± 234 vg of BEV-derived rAAV6 (Figure 5A). A similar pattern was seen in the liver: 9120 ± 2456 vg in HEK293-derived rAAV6 treated mice livers compared to 2297 ± 848 vg in BEV derived rAAV6 treated mice livers (Figure 5B).

Comparison was also made for transgene expression levels in heart and liver tissues. GFP expression in the heart was significantly higher in mice that received HEK293-derived rAAV6-GFP (451 ±1 58 fold) compared to mice that received BEV derived rAAV6-GFP (9 ± 2 fold) (Figure 5C). GFP expression was not significantly different in the liver between BEV (5583 ± 2492 fold) and HEK293-derived rAAV6-GFP (87,106 ± 29,606 fold) (Figure 5D). Thus, HEK293-derived rAAV6-GFP was not only better at entering the cell but was also capable of significantly (about 17-fold) higher expression as indicated by the expression index values in the heart (Figure 5E,F). In the liver, the expression index trended higher (approximately 3-fold) in HEK293-derived vector compared to BEV-derived rAAV. The reason for this organ-specific difference is unclear and likely relates to differences in the AAV structure (capsid/genome) and the resulting functional changes.

**Figure 6:**
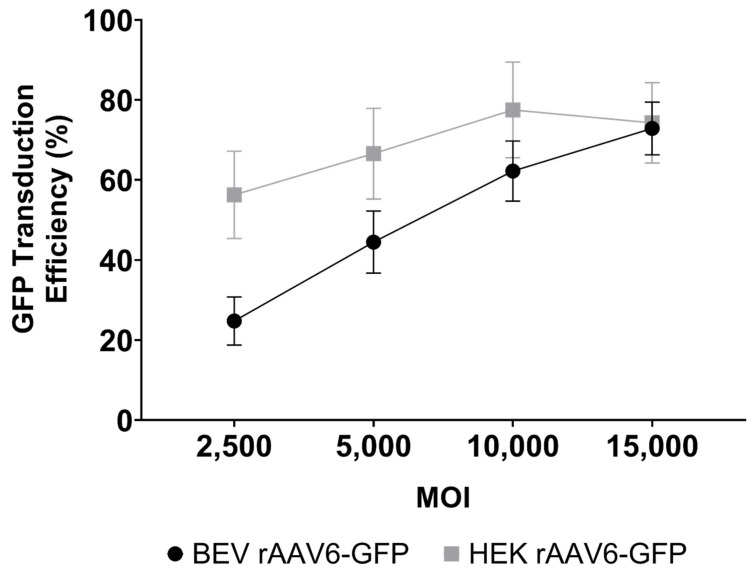
Dose response curve for GFP transduction of 2V6.11 cells at MOIs ranging from 2500 to 15,000 with either HEK293 or Sf9 derived rAAV6-GFP. At 48 h post transduction, cells were harvested, and the percentage of GFP-positive cells were determined via flow cytometry. The results are from three independent experiments displayed as mean ± SEM.

Although there was no difference in the transduction capabilities of HEK 293 and BEV derived rAAV6-GFP on cardiac cells in vitro (Figure 4), this was not replicated in vivo. Significantly higher copy numbers of vector genomes were seen in organs of mice that had received HEK293-derived rAAV6-GFP as compared to BEV derived vector. It was hypothesised that there may have been saturation of the GFP signal in vitro, since a high MOI of 30,000 was used and achieved a transduction rate of >75% (Figure 4). To help resolve this discrepancy, a dose response curve was performed to determine the transduction efficiencies for the BEV and HEK293-derived rAAV6-GFP preparations at lower MOIs ranging from 2500 to 15,000 in 2V6.11 cells (Figure 6). HEK293-derived rAAV6-GFP vectors showed significantly higher transduction efficiencies compared to BEV derived vector at MOIs up to 10,000 with no difference seen at the highest MOI of 15,000. The HEK293-derived rAAV6-GFP vectors showed a plateau beyond a MOI of 10,000 indicating transduction saturation.

This reduced relative transduction efficiency of BEV derived rAAVs at lower MOIs could explain the reduced transduction performance in vivo. The reason for lower transduction efficiencies for rAAV serotypes 8 and 9 in our system (Figure 2) can be explained by the lack of VP1 protein and the incorrect VP stoichiometry as previously reported [9,24] and demonstrated in Figure 3. Furthermore, a report published by Galibert et al., showed that cathepsin-induced degradation of VP1/VP2 proteins occurred in BEV derived rAAV8 [25]. However, we could not detect the supplementary degradation products as observed by Galibert et al., for BEV derived rAAV8.

For BEV derived rAAV6, we observed optimal VP protein stoichiometry (Figure 3). Since the cleavage site recognised by baculoviral cathepsin is also present in the rAAV serotype 6 capsid, we repeated the western blot on multiple preparations of BEV derived rAAV6 and imaged them using the more sensitive infrared LICOR system (Figure S3). We were unable to detect cathepsin degradation products in the rAAV6 preparations, thus discounting the possibility that cathepsin degradation was responsible for its reduced transduction efficiency.

One explanation for poor transduction efficiencies of BEV derived rAAV6 in the in vivo setting could be due to the larger percentage of empty versus filled capsids in BEV derived rAAV as opposed to the HEK293 preparations [26,27,28]. Since transduction efficiency is strongly dependent on the efficiency of the vector’s ability to enter cells and express the transgene of interest, a higher percentage of empty capsids decreases this efficiency. Since the expression cassette encoding GFP is similar in both systems, expression of the transgene should not be affected.

More recently, Tran et al. reported [29] that the BEV system produced a high degree of truncated and unresolved species of rAAV vector genomes as compared to the HEK system which, while not impacting PCR titre, may impair performance. As speculated by Tran et al., it is not known whether presence of mutant ITRs in BEV vector preparations can impact transgene expression and stability in cells.

A recent report has documented that rAAV capsids undergo post translational modifications (PTMs) such as glycosylation, acetylation, phosphorylation, and methylation. rAAV genomes were also found to be methylated. It was concluded that differences in PTMs between the human and insect production platforms was responsible for difference in potency of rAAV produced [30]. Such PTMs and genomic methylation may underpin the differences in performance observed in our study.

We have successfully produced BEV rAAV6-GFP using the 2 BAC system as described by Urabe et al. [6], wherein the start codon for VP1 was altered to allow for the correct stoichiometry of VP proteins. Although this change did not lead to differences in titres and allowed for the correct stoichiometry of VP proteins, the difference in transduction efficiency of the BEV derived rAAV as compared to the HEK293-derived rAAV could be due to composition of viral particles in terms of empty versus filled capsids or to changes in receptor binding, endocytosis, or intracellular pathways. Interestingly, the presence of ITR mutants, while unproven in our study, may also explain the reduced transduction efficiency of BEV derived AAV.

There are inherent and important limitations to our study that merit further work to elucidate the otherwise unexplained mechanism(s) for the observed difference in vector performance based on production method, including organ specific differences (heart versus liver). The relative abundance of empty versus full capsids is an important determinant of vector performance. Similarly, assessment of genomic integrity with next generation sequencing of vector genomes and an assessment for an array of possible PTMs or genomic methylation could also be helpful in informing the mechanism(s) for vector performance differences.

## 4. Conclusions

Our study demonstrates that rAAV bioactivity in cardiomyocytes is superior when vector is produced by the HEK293 method as compared to the BEV system. This functional discrepancy is not only dependent on the stoichiometry of VP proteins as demonstrated by wild type AAV, but there are also other properties of the vector capsid/genome that may be affected during production in the baculovirus/insect system that have a bearing on functional performance in vitro and in vivo in cardiomyocytes.

## Data Availability

Not applicable.

## Supplementary Materials

The following supporting information can be downloaded at: https://www.mdpi.com/article/10.3390/v14081623/s1. Supplementary methods, Figure S1: Schematic of protocol used to differentiate hiPSC WTC cell line into cardiomyocytes with small molecule modulators of canonical Wnt signalling, Figure S2: Flow gating strategy to identify GFP positive cells, Figure S3: 1 × 10^11^ vector genomes of two HEK293 and three Sf9 derived rAAV cap6 preparations were run on 4–12% Bis Tris polyacrylamide gel and western blotting using a monoclonal anti VP1 + VP2 + VP3 antibody was performed to detect Cathepsin induced degradation of VP proteins, Table S1: rAAV-GFP primer/probe sequences targeting the GFP element in the rAAV vector construct and Table S2: Mouse primer sequences targeting the GFP and GAPDH sequences in synthesised cDNA.
