# Supplementary material for: Performance of Cardiotropic rAAV Vectors Is Dependent on Production Method
Source: Viruses. 2022 Jul 26;14(8):1623. doi: 10.3390/v14081623 (PMC9341392; doi:10.3390/v14081623)
Supplement: Supplementary file 1 [file viruses-14-01623-s001.zip › viruses-1814712-supplementary.pdf]

Supplementary Methods

rAAV vector production in HEK293 cells

Briefly, rAAV 2/8 and rAAV 2/9 were produced using the 3-plasmid system consisting of AAV-CBA-GFP-WPRE plasmid, AAV-Cap 2/8 or Cap 2/9 packaging plasmid and pXX6 helper plasmid. rAAV 2/6 was produced using the two-plasmid system consisting of AAV-CBA-GFP-WPRE and the packaging/helper plasmid pDGM6. These plasmids were transfected into HEK293 cells by calcium phosphate precipitation. For rAAV 2/8 and rAAV 2/9 cells were collected while for rAAV 2/6, the supernatant was collected, 48 hours post transfection and stored at -80°C (cell pellet) or 4°C (clarified supernatant) until purification.

Vector was purified from the rAAV 2/8 and 2/9 cell pellet or rAAV 2/6 supernatant as previously described [14]. A final qPCR was performed against WPRE in the vector expression cassette, to determine the titre of the concentrated vector stocks. The final qPCR titres were ~1×10^{13} vg/mL for rAAV 2/8 and 2/9, and ~4×10^{12} vg/mL for rAAV 2/6.

rAAV Vector production in Sf9 cells

$2\text{x}10^6$ cells/mL of Sf9 cells were coinfected with either rBac-Rep2/Cap 6, 8 or 9 and rBac-GFP virus stocks at a ratio of 3:1 multiplicity of infection (MOI) with a total MOI of 0.12. Twenty-four hours post infection the temperature of the incubator was raised to 30°C. At 72 hours post infection, when the cell viability was ~30% – infected cells were collected by centrifugation at 300g for 10 minutes, and the culture medium retained for further processing. The infected cell pellet was resuspended in TNT extraction buffer (20mmol/L Tris–HCL [pH 7.5], 150mmol/L NaCl, 1% Triton X-100, 10 mmol/L MgCl2), and stored at -80°C. Purification of baculoviral derived rAAV8 and 9 was carried out as described for HEK293-derived rAAV8 and 9 using the ammonium sulphate precipitation protocol. Baculoviral rAAV6 infected cells were freeze thawed three times before the supernatant was benzonase treated at 37°C for 30 minutes. Post centrifugation the supernatant was mixed with the culture medium that was retained. Two percent PEG was added and incubated overnight with shaking. The pellet was collected by centrifugation at 4000rpm for 15 minutes at 4°C. The pellet was resuspended in cesium chloride and layered on a cesium chloride density gradient before being ultracentrifuged and further processed as described in the HEK293-derived rAAV purification protocol [12].

Neonatal rat ventricular myocyte (NRVM) isolation

Primary cultures of cardiac myocytes were prepared by enzymatic digestion of ventricles obtained from neonatal (2-3 day old) Sprague-Dawley rats. Briefly, ventricles (n = 8-12 per litter) were incubated with 0.1% trypsin in hanks balanced salt solution, overnight and dissociated in four to five sequential steps using 0.1% collagenase. Isolated cells were resuspended in culture medium (M199, supplemented with 10% FBS, 50 U/mL penicillin, Vitamin B12, NEAA, Glutamine and 10 mM HEPES) and pre-plated into T150 tissue culture flasks and incubated at 37°C for 2 hours to enrich for cardiomyocytes. A cell count was performed, and cells were plated at a density of $2\text{x}10^5$ cells/well in M199+10% FBS media.

Human induced-pluripotent stem cell-derived cardiomyocytes (hiPSC-CMs)

Briefly, Human WTC iPSCs were maintained on Geltrex coated plates in TeSR-E8 media. Standard cardiomyocyte directed differentiation using a monolayer platform was performed with a modified protocol based on previous reports [20, 21] (Figure S1Figure S1). On day -2 of differentiation, human WTC iPSCs were dissociated using 1x Tryple Express, plated onto Geltrex coated plates and cultured over 2 days in TeSR-E8 media until >97% confluent. Differentiation was induced on day 0 by changing the culture media to RPMI 1640 containing 6μM CHIR-99021, 1x B27-Insulin supplement and 1x penicillin-streptomycin solution. The next day, the media was replaced with RPMI containing 1x B27-Insulin and 1x penicillin-streptomycin solution. On day 3, the media was exchanged for RPMI containing 1x B27-Insulin, 1x penicillin-streptomycin and 5μM IWP-2. On day 5, the media was exchanged for RPMI containing 1x B27 supplement and 1x penicillin-streptomycin without supplemental cytokines. From day 5 onwards, the cultures were fed every 2 days with RPMI plus 1x B27 supplement and 1x penicillin-streptomycin. Cultures were then purified using a chemically defined DMEM media (no glucose, no glutamine, no phenol red) supplemented with 0.1mM Lactic acid, 0.1% BSA and 1x penicillin-streptomycin for 4-6 days to yield >80% cardiac troponin T positive cardiomyocytes.

Supplementary Figures

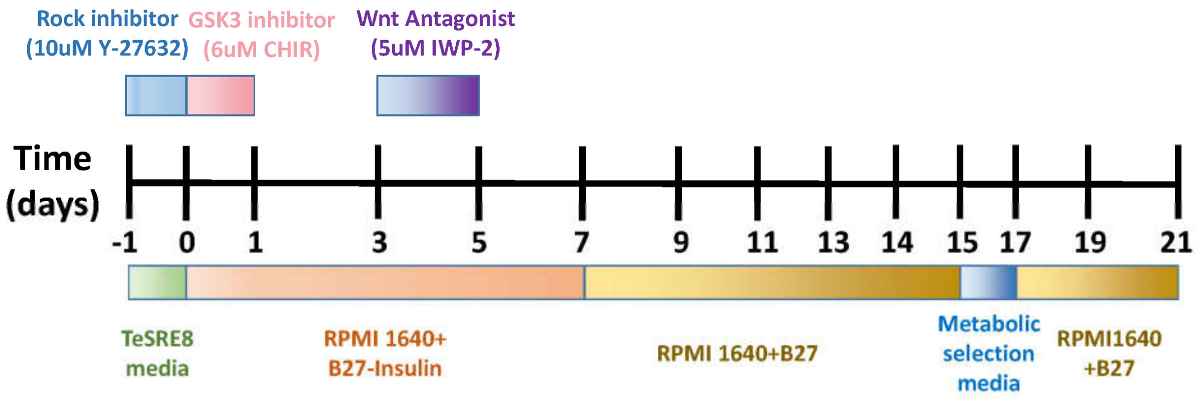

Figure S1: Schematic of protocol used to differentiate hiPSC WTC cell line into cardiomyocytes with small molecule modulators of canonical Wnt signalling.

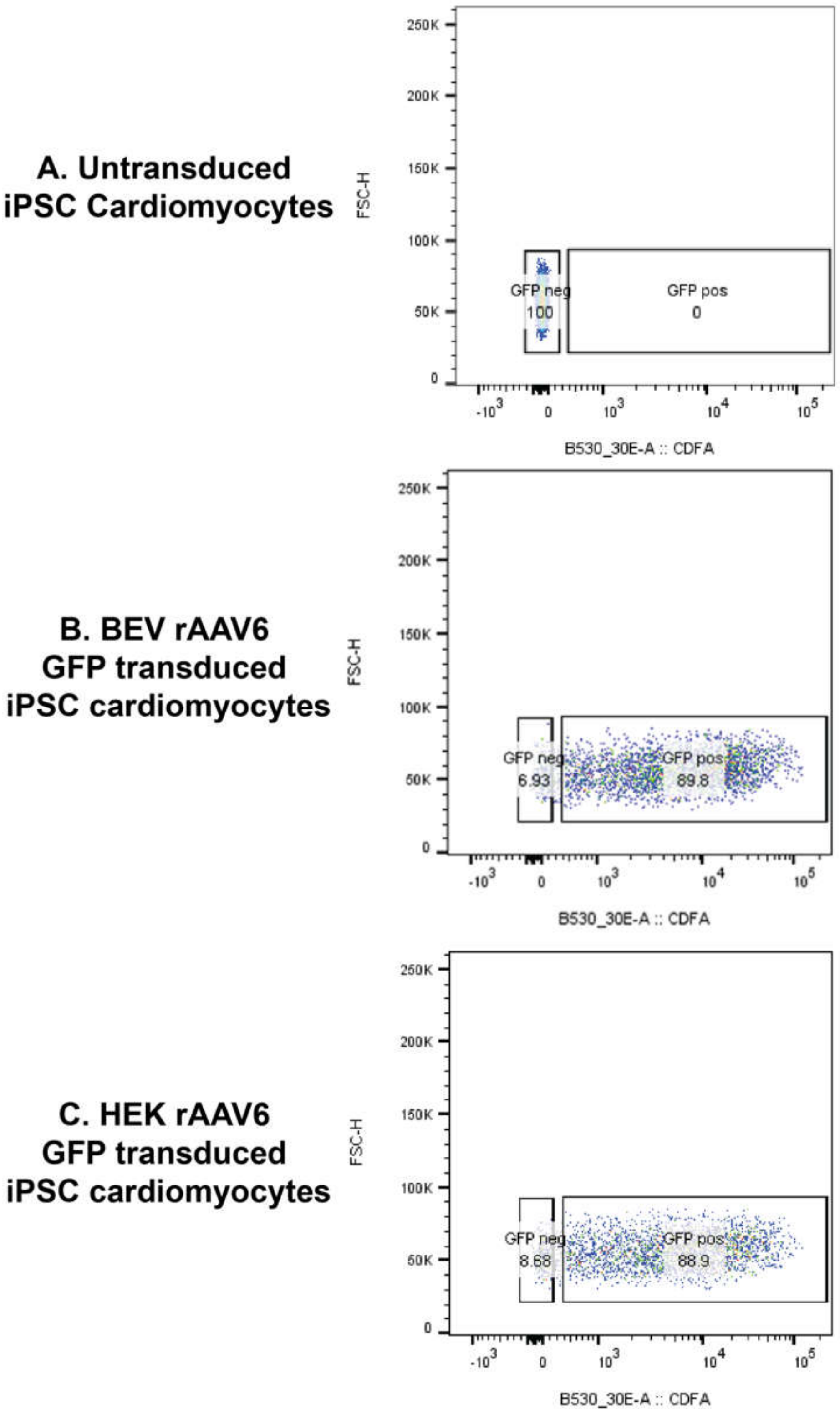

Figure S2: Flow gating strategy to identify GFP positive cells in (A) untransduced cells, (B) BEV rAAV6-GFP transduced iPSC cardiomyocytes and (C) HEK rAAV6-GFP transduced iPSC cardiomyocytes.

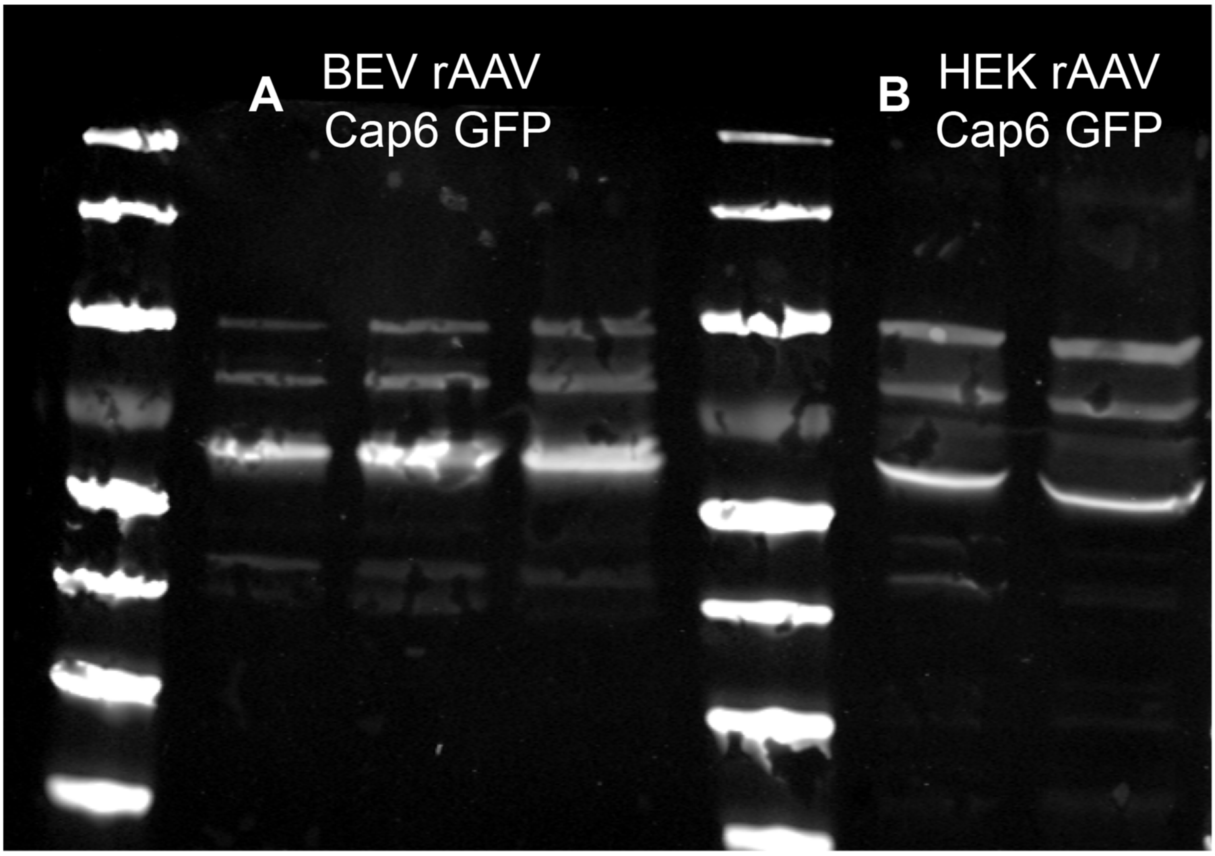

Figure S3: 1x10^{11} vector genomes of two HEK293 and three Sf9 derived rAAV cap6 preparations were run on 4-12% Bis Tris polyacrylamide gel and western blotting using a monoclonal anti VP1+VP2+VP3 antibody was performed to detect Cathepsin induced degradation of VP proteins. Imaging was done using the LICOR Odyssey system. This was to check for cathepsin degradation in rAAV6 Capsids using anti-VP Polyclonal antibody.

Supplementary Tables

Table S1: rAAV-GFP primer/probe sequences targeting the GFP element in the rAAV vector construct.

Name of Primer/Probe	Sequence
GFPF primer (forward)	5'-CTGCTGCCCGACAAC CAC-3'
GFPR primer (reverse)	5'-TCACGAACTCCAGCAGGA CT-3'
GFP probe	[6FAM] CCAGTCCGCCCTGAGCAAAGACC [TAM]

Table S2: Mouse primer sequences targeting the GFP and GAPDH sequences in synthesised cDNA.

Name of Primer/Probe	Sequence
qPCR GFP forward primer	5'-TCAAGATCCGCCACAACATC-3'
qPCR GFP reverse primer	5'-TTCTCGTTGGGGTCTTTGCT-3'
qPCR Rp1p0 forward primer	5'-ATGATGCGCAAGGCTATCAG-3'
qPCR Rp1p0 reverse primer	5'-CAGCAGCTGGCACCTTATTG-3'
